# Relationship between Inflammatory Markers and New Cardiovascular Events in Patients with Acute Myocardial Infarction Who Underwent Primary Angioplasty

**DOI:** 10.5539/gjhs.v5n4p48

**Published:** 2013-03-20

**Authors:** Eluisa La Franca, Marco Caruso, Angela Sansone, Rosanna Iacona, Laura Ajello, Dario Mancuso, Fabiana Castellano, Salvatore Novo, Pasquale Assennato

**Affiliations:** 1 Division of Cardiology, Department of Internal Medicine, Cardiovascular and Nephro-Urological Diseases, University Hospital “Paolo Giaccone” of the University of Palermo, Palermo, Italy

**Keywords:** C-reactive protein, myocardial infarction, primary angioplasty

## Abstract

**Introduction:**

The determination of inflammation markers in circulation has enabled an important improvement in the study of cardiovascular diseases. It was tested the hypothesis that non-specific markers such as erythrocyte sedimentation rate (ESR), C-reactive protein (CRP) and fibrinogen may provide prognostic information in patients with acute myocardial infarction with persistent ST-segment elevation (STEMI) undergoing primary angioplasty (PCI).

**Methods:**

Patients: A cohort of 197 consecutive patients with STEMI undergoing primary PCI was enrolled, evaluating during hospitalization, the peak values of the following markers of inflammation: ESR, CRP and fibrinogen. A telephone follow-up has been made in order to investigate any possible new cardiovascular events after hospital discharge and the procedure performed.

**Results:**

Higher values of CRP were statistically associated with adverse future events as composite endpoint and with the single endpoint of death. Furthermore, higher age, presence of hypertension, history of previous cardiovascular events, were statistically significantly associated with cardiac events at follow up. In this group were also overrepresented subjects with anterior myocardial infarction in the anterior localization and with an EF ≤ 35% at discharge.

**Conclusions:**

CRP appears to be a predictor of future cardiovascular events, confirming that a pro-inflammatory state promotes the progression of atherosclerotic disease and its complications.

## 1. Introduction

Atherosclerosis is a progressive and dynamic result of the complex interactions among endothelial dysfunction, inflammation (Ross, 1999a) and risk factors. The maintenance of vascular homeostasis depends on the balance between vasodilator and vasoconstrictor factors. When this balance is altered by a combination of the classical cardiovascular and inflammatory risk factors, blood vessels are susceptible to atheroma formation. The inflammation mediators (Ross, 1999b), in particular, support the evolution of the plaque and promote its rupture. Atheroma inflammation (Libby, 2001, 2002), in fact, leads to the tissue recruitment of T lymphocyte and macrophages (Glass & Witztum, 2001; Lusis, 2000), releasing enzymes and cytokines such as IL-6, which increases the plasma levels of fibrinogen and C-reactive protein (CRP) (Ramasamy, 2011). Among the inflammation markers, the CRP is a liver acute phase protein which produces multiple effects on endothelial biology, favouring a pro-inflammatory and pro-atherosclerotic phenotype. It stimulates ET-1 production, reduces the synthesis of nitroxide and increases several adhesion molecular expressions. The pro-inflammatory markers identification has involved a breakthrough in the study of cardiovascular diseases discriminating patients with increased risk of new events. Several studies (C. M. Albert, Ma, Rifai, Stampfer, & Ridker, 2002; Pearson et al., 2003) have shown that CRP, in particular, is an independent predictor of future events (myocardial infarction, restenosis after PCI and death) as well as providing additional information in the assessment of cardiovascular risk.

Once demonstrated the prognostic inflammation markers role, this cohort study is aimed to verify the hypothesis that non-specific markers as ESR, CRP and fibrinogen, could give prognostic indications in patients with acute myocardial infarction with persistent ST-segment elevation (STEMI), underwent primary angioplasty (PCI).

## 2. Methods

Eligible cases for this cohort study were 197 consecutive patients admitted to the Unit of Cardiology of the University of Palermo experiencing STEMI and underwent primary PCI. For all the considered patients the following data have been collected: demographic characteristics (age and sex); cardiovascular risk factors (hypertension, family history of vascular diseases, dyslipidemia, obesity, diabetes mellitus, smoke, moderate-to-severe renal insufficiency, assessed as a value of MDRD </_60ml/min); history of previous cardiovascular events (angina, myocardial infarction, TIA, stroke) and/or myocardial revascularization procedures (coronary angioplasty and aorto-coronary by-pass). Other characteristics of myocardial infarction have been considered as well, such as, sites (anterior, lateral, inferior), the peak of TnI and CK-MB during hospitalization, treatment (angioplasty with drug-eluting stent/metallic or with balloon only), post-procedure TIMI flow (thrombolysis in myocardial infarction) 3 or lower, the number of the vessels with critical stenosis (one 0>1) and, finally, the ejection fraction (EF%) of the left ventricle at discharge. During hospitalization seriated sampling of peripheral venous blood have been carried and peak values of markers of inflammation such as ESR, CRP and fibrinogen have been recorded. Finally, medical therapy at discharge was evaluated. We made a follow up on the phone in order to investigate about eventual new cardiovascular events after hospital admission and revascularization procedure, performed at our department. In the interviews were asked whether angina, re-infarction, new revascularization by angioplasty or by-pass, death has occurred. At the follow up, we also evaluated the home therapy and the patients compliance to it. The study deals also with patient's blood pressure, LDL cholesterol and glycemia (Lipsy, 2003). Finally, we compared the characteristics of the considered patients in question, considering first as composite endpoint cardiac events (angina, myocardial infarction and death), and after single endpoint of death.

### 2.1 Statistical Analysis

We used the ANOVA procedures in its simplest form in order to test the hypothesis that the means of several groups are all equal. The Student's t-test has been used to determine if two sets of data are significantly different from each other. In order to verify if proportions within groups are different, we used the chi-squared test. The statistical hypothesis tests have been implemented in a excel worksheet. Finally, we considered the p-value <0.05 as statistically significant.

## 3. Results

We enrolled 197 patients admitted to our department during the period between January 2004 and December 2009 because of acute myocardial infarction and underwent to primary PCI. After 42.5 months, cardiac events occurred in 27 patients (13.7%), death in 16 patients (8.1%). Tables from n.1 to n.3 compare patients with cardiac events at follow up (n=27) and controls (n=170). Between demographic and clinical characteristics, higher age (67.07 ± 11.2 v 61.1 ± 10.0; p<0.01), presence of hypertension (81.5% vs 62.9%; p=0.04), history of previous cardiovascular events (40.7% vs 19.6%; p=0.01), were statistically significantly associated with events at follow up (Table 1). In patients with events at follow up, were also overrepresented subjects with anterior myocardial infarction (66.7% vs 45.3%; p=0.03) and with an EF ≤ 35% at discharge (22.2% vs 4.1%; p<0.01) (Table 2). Concerning with the therapy at home among patients experiencing events, a smaller number of them practicing therapy with ASA (47.6% vs 97%; p<0.01), ACE-inhibitor/ARB (42.9% vs 82.2%; p<0.01), beta blocker (47.6% vs 82.2%; p<0.01) and statins (52.4% vs 88.2%; p<0.01) (Table 3). Tables from n.4 to n.6 compare patients with mortality at follow up (n=16) and controls (n=181). Finally, considering mortality as a single event, it was been revealed that demographic and clinical characteristics, higher mean age (72.3 ± 9.8 vs 61.0 ± 9.9; p=0.01), hypertension presence (87.5% vs 63.5%; p=0.04), a history of previous cardiovascular events (43.8% vs 17.2%; p=0.01), and a MDRD value <60ml/min(56.3% vs 19.6%; p<0.01) were statistically significantly associated with the event (Table 4). In these cases were also more represented, in a statisically significant way, subjects with anterior myocardial infarction (75% vs 45.9%; p<0.o1) and with a FE ≤ 35% (37.5% vs 3.9%; p<0.01) (Table 5). Concerning with the inflammation markers, an average of a higher CRP level is finally resulted, in a statistically significant way, associated with death (7.76 ± 11.8 vs 2.46 ± 3.3; p<0.01) (Table 6).

**Table 1:** Demographic and clinical characteristics about population with and without cardiac events at follow up. MDRD (Modification of Diet in Renal Disease)

Demographic and clinical characteristics			
Total sample n= 197			
	No Cardiac Events (n=170)	Cardiac Events (n=27)	P
Age± σ	61.1 ± 10.05	67.07 ± 11.26	P=<0.01
Sex n %	M 139(81.8) F 31(18.2)	M19(70.4) F 8(29.6)	NS
Family hystory n %	50 ( 29.4)	9 (33.3)	NS
Hypertension n %	107 (62.9)	22 (81.5)	P=0.04
Dyslipidemia n %	78(45.9)	7 (25.9)	P=0.03
Obesity n %	43 (25.3)	6 (22.2)	NS
Diabetes n %	46 (27.1)	8 (29.6)	NS
Smoke n %	107(62.9)	9 (33.3)	P=<0.01
Previous Events n %	27 (16.0)	11(40.7)	P=<0.01
MDRD<60 ml/min n %	33 (19.6)	11 (40.7)	P=0.01

**Table 2:** Demographic and clinical characteristics about population with and without cardiac events at follow up

Characteristics infarction related			
Total sample n= 197			
	No Cardiac Events (n=170)	Cardiac Events (n=27)	P
Anterior infarction n %	77 (45.3)	18 (66.7)	P=0.03
BMS treatment n %	134 (78.8)	20 (74.1)	NS
DES treatment n %	17 (10.)	3 (11.1)	NS
POBA treatment n%	14 (8.2)	1 (3.7)	NS
Unsuccessful PCI n %	5 (2.5)	3 (1.5)	NS
TIMI flow<3 n%	23 (13.5)	5 (18.5)	NS
Obstructed vessels>1 n %	73(42.9)	16 (59.3)	NS
EF ≤35% n %	7 (4.1)	6 (22.2)	P=0.003
CK-MB ± σ	138.3. ± 104.7	160.9 ± 136.3	NS
TnI ± σ	42.6 ± 34.9	44.2 ± 37.0	NS

BMS (bare metal stent); DES (drug eluting stent); POBA (Plain Old Balloon Angioplasty); PCI (Percutaneous coronary intervention); post-procedure TIMI flow (thrombolysis in myocardial infarction grade flow); EF (ejection fraction)

**Table 3:** Characteristics about home therapies among population with and without cardiac events at follow up

Characteristics about home therapy			
Total sample n=190			
	No Cardiac Events (n=169)	Cardiac Events (n=21)	P
ASA n %	164(97.0)	10(47.6)	P<0.01
Clopidogrel n %	28(16.6)	2(9.5)	NS
ASA+Clopidogrel n %	21(12.4)	0(0.0)	NS
ACEI/ARB n %	139(82.2)	9(42.9)	P=0.0002
B-blocker n %	139(82.2)	10(47.6)	P=0.0009
Diuretic n %	31(18.3)	5(23.8)	NS
Nitrates n %	6(3.6)	1(4.8)	NS
Statins n %	149(88.2)	11(52.4)	P=0.0002
Oral hypoglycemic agents/Insulin n %	45(26.6)	3(14.3)	NS

ASA (acetylsalicylic acid); ACEI (Angiotensin Converting Enzyme Inhibitor); ARB (Angiotensin Receptor Blockers)

**Table 4:** Demographic and clinical characteristics about dead and not dead population at follow up. MDRD (Modification of Diet in Renal Disease)

Demographic and clinical characteristics			
Total sample n= 197			
	No Death (n=181)	Death (n=16)	P
Age± σ.	61.0 ± 9.9	72.3 ± 9.8	P = < 0.01
Sex n %	M 147(81.2) F 34(18.8)	M 11 (68.8) F 5(31.3)	NS
Family history n %	55 ( 30.4)	4 (25.0)	NS
Hypertension n %	115 (63.5)	14 (87.5)	P = 0.04
Dyslipidemia n %	84 (46.4)	1 (6.3)	P = < 0.01
Obesity n %	46 (25.4)	3 (18.8)	NS
Diabetes n %	49 (27.1)	5 (31.3)	NS
Smoke n %	111 (61.3)	5 (31.3)	P = < 0.01
Previous Events n %	31 (17.2)	7(43.8)	P = 0.01
MDRD<60 ml/min n %	35 (19.6)	9 (56.3)	P = < 0.01

**Table 5:** Dead and not dead population infarction related characteristics

Characteristics infarction related			
Total sample n = 197			
	No Death (n=181)	Death (n=16)	P
Anterior infarction n %	83 (45.9)	12 (75)	P = < 0.01
BMS treatment n %	143 (79.0)	11 (68.8)	NS
DES treatment n %	18 (9.9)	2 (12.5)	NS
POBA treatment n%	14 (7.7)	1 (6.3)	NS
Unsuccessful PCI n %	6 (3.0)	2 (1.0)	NS
TIMI flow<3 n%	24 (13.3)	4 (25.0)	NS
Obstructed vessels>1 n %	80(44.2)	9 (56.3)	NS
EF ≤35% n %	7 (3.9)	6 (37.5)	P = < 0.01
CK-MB ± σ.	137.1 ± 102.7	189.6 ± 163.2	NS
TnI ±	42.4 ± 34.6	46.6 ± 40.9	NS

BMS (bare metal stent); DES (drug eluting stent); POBA (Plain Old Balloon Angioplasty); PCI (Percutaneous coronary intervention); post-procedure TIMI flow (thrombolysis in myocardial infarction grade flow); EF (Ejection Fraction)

**Table 6:** Levels of inflammation markers in dead and not dead population at follow up

Characteristics inflammation markers related			
	No Death (n=181)	Death (n=16)	
Fibrinogen ± σ (n=197)	420.3 ± 132	477.0 ± 166.1	NS
	No Death (n=101)	Death (n=6)	
PCR ± σ (n=107)	2.46 ± 3.3	7.7 ± 11.8	P= <0.01
	No Death (n=35)	Death (n=1)	
ESR ± σ (n=36)	23.5 ± 20.5	11.0 ± 0.0	NS

## 4. Discussion

In this study some associations, according with data published in literature have emerged; others are probably influenced by sample size and by the lack of availability of some data, especially those related to inflammation markers concerning with the entire study population. Our data have confirmed the importance of age (Maggioni et al., 1993) as one of the major risk factors for cardiovascular events. Also the significant relationship between hypertension and new events is interesting (Bonow, Mann, Zipes, & Libby, 2011). About the ways in which the hypertension may influence cardiovascular events, it is known that emodynamics, metabolic and humoral factors are involved. Furthermore hypertension, acting also on the entire vascular tree, has an impact not only on heart but also on brain and kidney. Also the relationship between cardiac events (angina, myocardial infarction and death) and MDRD values<60ml/min was significant, corresponding to a state from moderate-to-severe renal insufficiency. The increased prevalence of coronary artery disease during Chronical Renal Insufficiency (CRI) is due to the presence of “traditional” risk factors and other “non-traditional” (Weiner et al., 2008). These last are represented by anemia, hyperphosphoremy as well as from a condition of micro-inflammation present at any stage of CRI, but it is accentuated in patients on haemodialysis. In uremic patients, in fact, there is a wide variety of factors capable of stimulating the monocyte and macrophage system and cytokines (vascular access infection, persistent asymptomatic infections). Concerning with this aspect it has been shown (Parfrey & Foley, 1999; Yeun, Levine, Mantadilok, & Kaysen, 2000; Zimmermann, Herrlinger, Pruy, Metzger, & Wanner, 1999) that this inflammatory state stimulates an increase of IL-6 and CRP, which may influence the progression of atherosclerotic disease (Oudi et al., 2010). The reduced kidney clearance of CRP and/or cytokines, may also play an important role in increasing of serum values and in the maintenance of chronic inflammatory status in these patients. Another factor associated with the follow up is a history of cardiovascular events. It is understandable that a history of infarction as well as stroke, especially if early, have a negative impact on prognosis. Other significant relationships emerged from this study are related to the infarction characteristics and in particular to the anatomopathological and functional conditions. The anterior localization, a severe impairment of ventricular function (EF<35%), the vessels’ number with critical stenosis (Sorajja et al., 2007) (>1) are associated with a worse prognosis. Concerning with this last factor, it has been shown that the success of the reperfusion treatment is heavily influenced by the presence of a multivessel disease: for this reason future studies aimed of optimizing the management of these patients are necessary. Concerning with the inflammation markers it should be noted that the average values of fibrinogen and CRP were higher of the ones associated to control cases. However, these differences did not reach the statistical significance that has emerged, instead, considering only death as an event. Primary prevention trials (Corrado & Novo, 2005, 2007) have demonstrated that CRP is an independent (Coppola et al., 2006; Corrado et al., 2006) risk factor triggering with the future cardiovascular events (Corrado, Rizzo, Muratori, Coppola, & Novo, 2008). However, if on one hand blood pressure, smoke or LDL cholesterol serum levels are recognized as risk factors, it is not yet demonstrated that CRP is a risk factor and therefore it has not been defined so strict its additional value in the current risk scores. The Study could add information about the role of these markers as indicators of risk in secondary prevention. Finally, in therapy practice, we found in our study that among patients experiencing events, fewer practicing therapy with ASA, ACE-inhibitor/ARB, beta blockers and statins. It is therefore evident the importance of secondary prevention therapy in acting at multiple levels (Miedema et al., 2012) such as platelet aggregation, blood pressure control, left ventricular remodeling, LDL cholesterol levels in the blood and anti-inflammatory, ASA (Hennekens, Buring, Sandercock, Collins, & Peto, 1989), ACE inhibitors (Yusuf et al., 2000) and statins mediated. Recently it has been paid particular attention to the pleiotropic effects of statins, resulting not only from lipid-lowering but also by anti-inflammatory properties (Birnbaum & Ye, 2012). There are numerous studies showing that statins reduce the CRP levels and the benefits they bring in individuals with inflammation evidence even in absence of cardiovascular diseases and/or dyslipidemia (Ridker, Rifai, Pfeffer, Sacks, & Braunwald, 1999; Ridker et al., 1998).

Among patients with acute myocardial infarction underwent to primary PCI, the older subjects with hypertension, with renal dysfunction from moderate-to-severe and with positive history of previous cardiovascular events, show a greater risk of events at follow up. The anterior localization, the multivessel disease and an ejection fraction less than 35% have a negative impact on prognosis. Concerning with the inflammation markers it has been confirmed the role of CRP in particular as a predictor of future cardiovascular events at follow up, that in our study is death. These data confirm that, in addition to the known clinical and demographic factors, also a pro-inflammatory status influences the progression of atherosclerotic disease and its complications. This is demonstrated indirectly by the positive drugs effects in secondary prevention which also extent an anti-inflammatory effect (M. A. Albert, Danielson, Rifai, & Ridker, 2001).
